# Alcohol SBIRT implementation in adult primary care: physician versus non-physician delivery

**DOI:** 10.1186/1940-0640-8-S1-A49

**Published:** 2013-09-04

**Authors:** Jennifer Mertens, Stacy Sterling, Constance Weisner, David Pating

**Affiliations:** 1 Kaiser Permanente Division of Research, Oakland, CA, USA; 2 Department of Psychiatry, University of California, San Francisco, San Francisco, CA, USA

## 

Alcohol screening, brief intervention, and referral to treatment (SBIRT) in adult primary care is cost-effective and recommended by national guidelines, but has not been widely adopted. The ADVISE study examines whether non-physician (versus physician) delivery of SBIRT in primary care clinics increases implementation. This is a clustered, randomized controlled trial of 54 adult PC clinics in Kaiser Permanente Northern California. We compared a control condition versus training, technical support and performance feedback for physician versus non-physician delivery. The study included qualitative interviews with providers and patients and examined rates of screening, brief intervention and referral as implementation outcomes. On average, interventions were longer in duration in the non-physician arm, but barriers in this model included a complex ‘handoff”, the need for patients to often have a separate visit for the intervention, and patient resistance. Barriers salient for physician delivery included a region-wide primary care access initiative and new competing priorities due to health care reform. Inclusion of the screener in the EMR, opinion leaders, a physician testimonial linking alcohol use to performance measures for other chronic conditions, and compatibility with other practices were facilitators. We discuss findings in the context of the Consolidated Framework on Implementation Research.

